# P-1177. The Single-Center Descriptive Analysis for the Efficacy and Adverse Reactions of the All-oral Short-term Regimen Containing Contezolid in the Treatment of Patients with Multidrug-resistant/Rifampicin-resistant Pulmonary Tuberculosis

**DOI:** 10.1093/ofid/ofaf695.1370

**Published:** 2026-01-11

**Authors:** Yuxia Zhang, Yu Xiong, Tingting Chang, Yanming Sun, Zhiyuan Zhao, Fengxia Liu

**Affiliations:** Department of Tuberculosis VII, Shandong Public Health Clinical Center Affiliated to Shandong University, Jinan, Shandong, China (People's Republic); Department of Tuberculosis VII, Shandong Public Health Clinical Center Affiliated to Shandong University, Jinan, Shandong, China (People's Republic); Department of Tuberculosis VII, Shandong Public Health Clinical Center Affiliated to Shandong University, Jinan, Shandong, China (People's Republic); Department of Tuberculosis VII, Shandong Public Health Clinical Center Affiliated to Shandong University, Jinan, Shandong, China (People's Republic); Department of Tuberculosis VII, Shandong Public Health Clinical Center Affiliated to Shandong University, Jinan, Shandong, China (People's Republic); Shandong Public Health Clinical Center Affiliated to Shandong University, Jinan, Shandong, China

## Abstract

**Background:**

To explore the efficacy and safety of the 6-month all-oral short-term regimen containing contezolid in the setting of the multidrug-resistant/rifampicin-resistant pulmonary tuberculosis.

**Table 1.**
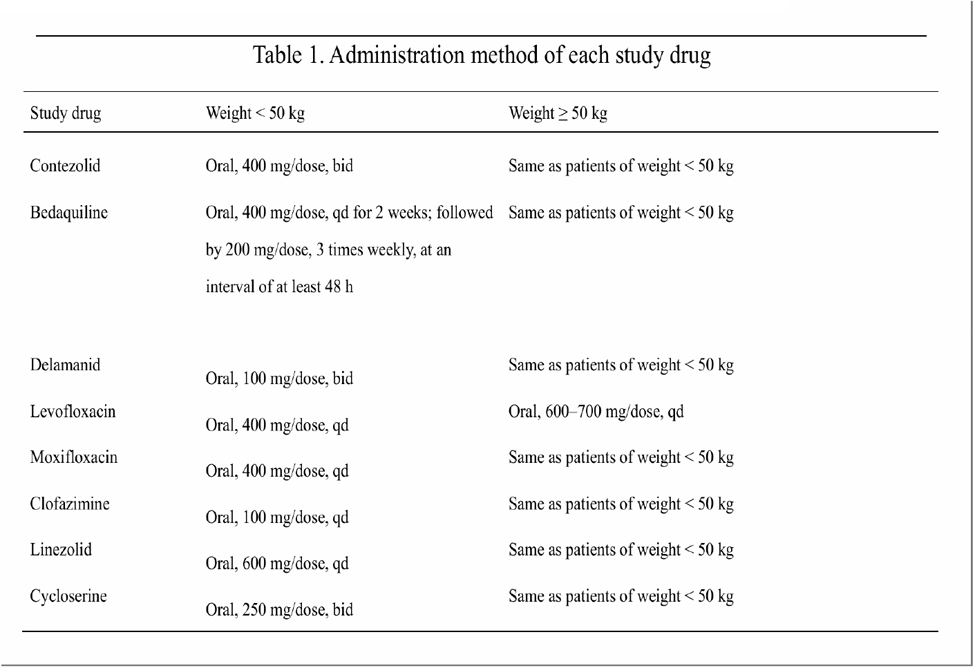
Administration method of each study drug

**Table 2.**
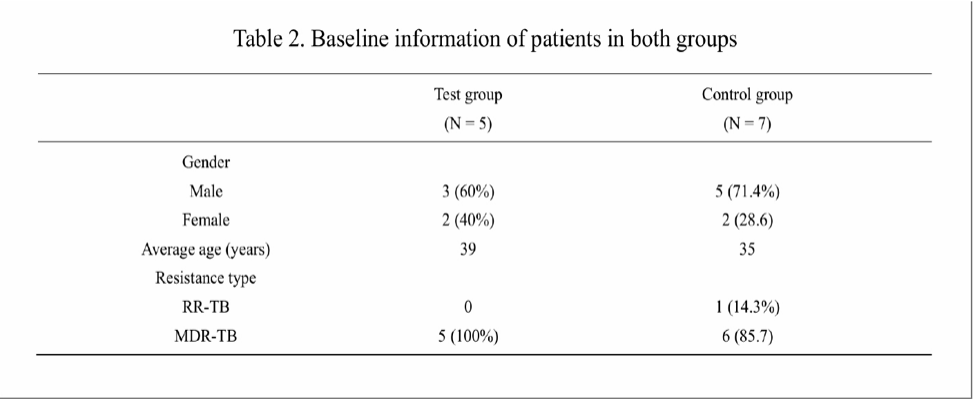
Baseline information of patients in both groups

**Methods:**

The study subjects were adult patients with multidrug-resistant/rifampicin-resistant pulmonary tuberculosis, randomized into the test and control group. The subjects in the test group were given contezolid + bedaquiline + delamanid + fluoroquinolones for 6 months, and the subjects in the control group were given 18-month WHO standard-of-care regimen. The specific administration methods are shown in Table 1. The primary study endpoint was the sputum negative conversion rate and the incidence of adverse reactions after 6 months of treatment.

**Results:**

A total of 12 eligible patients were enrolled, including 5 patients in the test group and 7 patients in the control group. The baseline data are shown in Table 2. The patients in both groups completed the 6-month treatment, and all patients achieved negative conversion of sputum acid fast bacillus smear and sputum tuberculosis culture. The median time to negative conversion of sputum smear and sputum culture was 4 weeks (2 weeks, 16 weeks) and 4 weeks (4 weeks, 8 weeks) in the test group, and 4 weeks (2 weeks, 12 weeks) and 4 weeks (4 weeks, 8 weeks) in the control group, respectively. During the treatment, 2 (40%) patients in the test group experienced drug-related adverse events, mainly manifested as aspartate aminotransferase/alanine aminotransferase increased, headache, and dizziness; 7 (100%) patients in the control group experienced drug-related adverse events, including QTc interval prolonged in 3 patients, anemia and white blood cell decreased in 2 patients, aspartate aminotransferase/alanine aminotransferase increased in 1 patient, and visual acuity decreased in 1 patient. The incidence of adverse reactions in the control group was significantly higher than that in the test group (p = 0.022). See Table 3 and Table 4. None of the patients experienced serious adverse events.

**Conclusion:**

This study suggested that the all-oral short-term regimen containing contezolid was comparable to the traditional long-term regimens in sputum negative conversion rate after 6 months of treatment, with decreased incidence of adverse reactions.

**Disclosures:**

All Authors: No reported disclosures

